# Study protocol: type II hybrid effectiveness-implementation study of routine functional status screening in VA primary care

**DOI:** 10.1186/s43058-025-00698-w

**Published:** 2025-01-31

**Authors:** Francesca M. Nicosia, Kara Zamora, LauraEllen Ashcraft, Gregory Krautner, Marybeth Groot, Bruce Kinosian, Cathy C. Schubert, Sumedha Chhatre, Helene Moriarty, Orna Intrator, Andrea Wershof Schwartz, Ariela R. Orkaby, Jason Prigge, Rebecca T. Brown

**Affiliations:** 1 Center for Data to Discovery to Delivery Innovation (3DI), San Francisco Veterans Affairs (VA) Healthcare System, San Francisco, CA USA; 2 Institute for Health & Aging, School of Nursing, University of California, San Francisco, CA USA; 3 San Francisco VA Healthcare System, San Francisco, CA USA; 4 Center for Health Equity Research and Promotion, Corporal Michael J. Crescenz VA Medical Center, Philadelphia, PA USA; 5 Department of Biostatistics, Epidemiology, and Informatics, Perelman School of Medicine, University of Pennsylvania, Philadelphia, PA USA; 6 Leonard Davis Institute of Health Economics, University of Pennsylvania, Philadelphia, PA USA; 7 Central Office of Geriatrics and Extended Care, District of Columbia, Washington, USA; 8 Geriatrics & Extended Care Data & Analyses Center (GECDAC), Canandaigua VAMC, Canandaigua, NY USA; 9 Geriatrics and Extended Care Program, Corporal Michael J. Crescenz VA Medical Center, Philadelphia, PA USA; 10 Division of Geriatric Medicine, Department of Medicine, University of Pennsylvania Perelman School of Medicine, Philadelphia, PA USA; 11 Community, Home, and Geriatrics Service, Richard L. Roudebush VA Medical Center, Indianapolis, IN USA; 12 Division of General Internal Medicine and Geriatrics, Indiana University School of Medicine, Indianapolis, IN USA; 13 Department of Psychiatry, Perelman School of Medicine, University of Pennsylvania, Philadelphia, PA USA; 14 Nursing Service, Corporal Michael J. Crescenz VA Medical Center, Philadelphia, PA USA; 15 M. Louise Fitzpatrick College of Nursing, Villanova University, Villanova, PA USA; 16 Department of Public Health Sciences, University of Rochester Medical Center, Rochester, NY USA; 17 New England Geriatric Research Education and Clinical Center (GRECC), VA Boston Healthcare System, Boston, MA USA; 18 Department of Epidemiology, T.H. Chan School of Public Health, Harvard University, Cambridge, MA USA; 19 Division of Aging, Brigham & Women’s Hospital, Harvard Medical School, Boston, MA USA

**Keywords:** Functional status, Frailty, Primary Care, Geriatrics, Implementation Science, Veterans, Veterans Health Administration

## Abstract

**Background:**

Maintaining functional status, defined as the ability to perform daily activities such as bathing, dressing, and preparing meals, is central to older adults’ quality of life, health, and ability to remain independent. Identifying functional impairments – defined as having difficulty or needing help performing these activities – is essential for clinicians to provide optimal care to older adults, and on a population level, understanding function can help anticipate service needs. Yet uptake of standardized measurement of functional status into routine patient care has been slow and inconsistent due to the burden posed by current tools. The goal of the Patient-Aligned Care Team (PACT) Functional Status Screening Initiative is to implement and evaluate a patient-centered, low-burden intervention to improve identification and management of functional impairment among older veterans in Veterans Health Administration (VHA) primary care settings.

**Methods:**

We will conduct a hybrid type 2 implementation-effectiveness cluster-randomized adaptive trial at 8 VHA sites using the Practical, Robust Implementation and Sustainability Model (PRISM) to guide implementation and evaluation. During a Pre-Implementation phase, we will engage clinical partners and develop local adaptations to maximize intervention-setting fit. During an Implementation phase, we will launch a standard bundle of implementation strategies (coalition building, champions, technical assistance) and system-level audit and feedback, identify sites with low uptake, and randomize those sites to receive continued standard vs. enhanced strategies (standard strategies plus clinician-level audit and feedback). The primary implementation outcome is reach (proportion of eligible patients at each site who receive screening/assessment) and the primary effectiveness outcome is appropriate management of impairment (proportion of patients with identified impairments who receive related referrals).

**Discussion:**

Implementing routine measurement of functional status in primary care has the potential to improve identification and management of functional impairment for older veterans. Improved management includes increasing access to services and supports for veterans and family caregivers, reducing potentially preventable acute care utilization, and allowing veterans to live in the least restrictive setting for as long as possible. Implementation will also provide data to inform the delivery of proactive interventions to prevent and delay development of functional impairment and improve quality of life, health, and independence.

**Trial registration:**

Registered at ClinicalTrials.gov on May 7, 2024, at NCT06404970 (https://clinicaltrials.gov/).

**Reporting guidelines:**

Standards for Reporting Implementation Studies (Additional file 1).

**Supplementary Information:**

The online version contains supplementary material available at 10.1186/s43058-025-00698-w.

Contribution to the literature

- Maintaining functional status, or the ability to perform daily activities such as bathing, dressing, and preparing meals, is central to older adults’ quality of life, health, and ability to remain independent, and identifying functional impairments is essential for clinicians to provide optimal care to older adults.
- The Patient-Aligned Care Team (PACT) Functional Status Screening Initiative is an evidence-based, patient-centered, low burden intervention to increase identification and improve management of functional impairment among older adults.
- Studies have not evaluated the adoption of routine, standardized functional status screening within a large national health care system.
- This study will evaluate the implementation of the PACT Functional Status Screening Initiative across 8 VA sites to assess (1) Reach (percentage of eligible patients who are screened and assessed for functional impairment) and (2) proportion of patients with identified impairment who receive appropriate care.

## Background

Measuring functional status, defined as the ability to perform daily activities such as bathing, dressing, or preparing meals, is essential to improve care and outcomes for older adults [1–8]. Older adults who lose independence in these activities have lower quality of life and a 2- to 3-fold higher risk of hospitalization, long-term institutionalization [9, 10], and death [8, 11]. Functional impairment, defined as having difficulty or needing help with daily activities, also strongly predicts caregiving needs and costs [10]. Proactively identifying functional impairment allows clinicians to deliver evidence-based interventions, such as physical and occupational therapy, to help delay or prevent nursing home admission [1–3, 12]. Understanding functional status is also essential to deliver patient-centered care, including evaluating how patients will tolerate interventions [4–6, 13, 14], individualizing screening and treatment for varied conditions [7, 8], assessing prognosis [8, 15, 16], and determining the need for long-term services and supports [17]. On a population level, health systems can use data about function to anticipate service needs [12, 18, 19]. For these reasons, clinicians and policy makers have called for standardized functional status measurement among older adults for decades [18, 20, 21].

Functional impairment is common among older veterans enrolled in the Veterans Health Administration (VHA), with an estimated 459,000 of 2.8 million veterans ages 65 and older experiencing impairment [10, 22, 23]. In 2022, VA expenditures for veterans requiring long-term care in nursing homes were an estimated $5.1 billion, which is projected to increase to $7.2 billion by 2037 [24]. To improve care and outcomes for older veterans with functional impairment, the VA Central Office of Geriatrics and Extended Care (GEC) has led efforts to integrate functional status measurement into primary care. In 2009, GEC implemented annual functional status screening among veterans ages 75 and older in primary care using an electronic tool administered during patient triage [25]. Measurement focused on the veteran’s ability to perform activities of daily living (ADLs; bathing, dressing, transferring, toileting, eating) and instrumental ADLs (IADLs; shopping, preparing food, managing medications, managing finances, doing housework, using transportation, using the telephone) [26, 27]. A national evaluation by our team later showed that screening uptake was low and of varying quality [25, 28, 29]. Moreover, when screenings were completed, the data collected were seldom used to inform patient care [25, 28, 29]. These findings pointed to the need for formal assessment of barriers to and facilitators of functional status measurement and data utilization and for more substantial input from patients and clinicians on design and implementation.

To improve identification and management of functional impairment, our team partnered with GEC to develop and pilot test a patient-centered, low-burden intervention in VHA Patient Aligned Care Teams (PACT; VHA’s version of Patient Centered Medical Home) (QUE 15–283). Our formative evaluation identified key barriers to and facilitators of routine measurement in primary care settings, including time limitations, competing priorities, and cumbersome electronic tools [29, 30]. Based on these findings, we developed a multi-component intervention for routine, standardized functional status measurement to facilitate increased identification and improved management of functional impairment among older patients. This includes annual screening by nurses and follow-up assessment by primary care providers, facilitated by electronic tools and templates and interprofessional education. In pilot testing in two primary care settings, implementation led to screening of 100% of 959 eligible veterans, with high rates of satisfaction among patients and primary care clinicians [31]. The success of the pilot study led to the development of the PACT Functional Status Screening Initiative, a research-operations partnership to implement and evaluate this initiative funded by the Department of Veterans Affairs (VA) Quality Enhancement Research Initiative (QUERI). Supported by GEC, VA Central Office of Primary Care, and VA Central Office of Nursing Services, the initiative aims to test the effectiveness of routine measurement and different implementation strategies across eight VHA sites to inform national roll-out.

### Study goals and objectives

The goals of the PACT Functional Status Screening Initiative are four-fold: (1) measure clinician- and organization-level implementation outcomes; (2) compare the effectiveness of a standard versus enhanced implementation approach to improve adoption; (3) measure patient-level clinical effectiveness of the intervention; and (4) test the effectiveness of electronic health record (EHR)-based frailty screening for identifying veterans at risk for functional impairment.

We will evaluate this initiative by conducting a hybrid type II implementation-effectiveness cluster-randomized adaptive trial [32–34]. During implementation, we will identify sites with low uptake and compare a standard bundle of implementation strategies to an enhanced bundle [35]. Based on our preliminary work [31], we hypothesize that this will lead to increased identification and improved management of functional impairment while providing data to inform VA strategic planning related to long-term services and supports and implementation of new programs. Specifically, we will generate data to guide VA’s investment in non-institutional care programs (including home- and community-based services) that can enhance veterans’ ability age in place. The findings will also allow for targeting and testing of novel programs to address impairments and prevent or delay further functional decline and associated acute care utilization.

### Conceptual framework and theoretical foundation

**Fig. 1:**
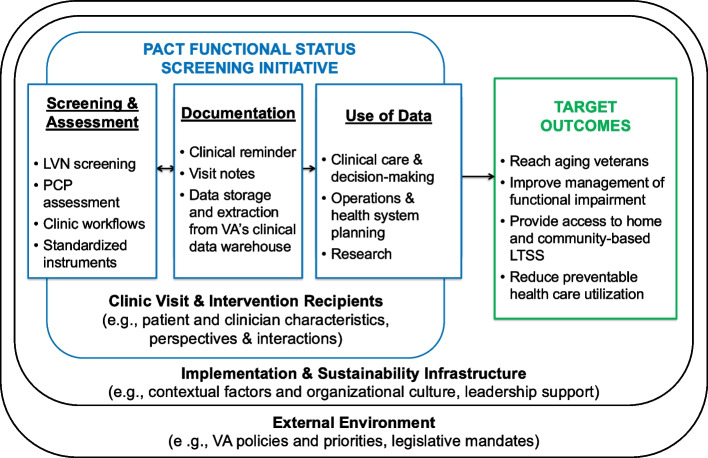
Conceptual Model for Functional Status in Primary Care Settings. The figure illustrates the relationship between processes of functional status measurement (screening and assessment, documentation, use of data), patient and clinician characteristics, contextual factors in implementation and sustainability infrastructures, and the external environment. These processes and structures influence target downstream outcomes. LVN indicates licensed vocational nurse, LTSS indicates Long-Term Services and Supports, PACT indicates Patient Aligned Care Team, PCP indicates primary care provider, and VA indicates Department of Veterans Affairs

Our conceptual framework for routine measurement of functional status in primary care, shown in Fig. 1, is informed by our prior formative evaluation [29] and the Practical, Robust Implementation and Sustainability Model (PRISM) [36, 37]. It illustrates the relationship between the processes of functional status measurement (screening and assessment, documentation, use of data), patient and clinician characteristics, contextual factors in implementation and sustainability infrastructures, and the external environment that includes VA priorities, policies, and legislative mandates (e.g., providing access to home- and community-based long-term services and supports VA’s Aging in Place Initiative and the 2022 Cleland Dole Act [38]). In turn, these processes and structures influence target downstream outcomes, including identification and management of functional impairment, healthcare utilization, and the ability to age in place. We will also use Normalization Process Theory (NPT) to guide qualitative data collection [39, 40]. NPT complements PRISM and considers the intervention itself, how staff understand the intervention, attitudes that develop around the intervention, and how it fits into existing roles, systems, and norms. NPT constructs include coherence, cognitive participation, collective action, and reflexive monitoring.

### Partnership approach

QUERI Partnered Evaluation Initiatives (PEIs) evaluate programs or policies that align with national VA priorities and have the potential to significantly impact healthcare for veterans. QUERI PEIs are jointly funded by QUERI and by operations partners. As detailed below, our project is partnered with several VA operational offices and overseen by a Technical Expert Panel. In addition, an Implementation Team and an Evaluation team comprised of both research and clinical operations team members will conduct the project. This team structure will allow for multi-disciplinary partnership and collaboration between research and operational groups.

#### Operational partners

Our operational partners include leaders from three national VA program offices: Geriatrics and Extended Care, Office of Primary Care, and Office of Nursing Services. In addition to these program leads we have enlisted multiple advisors and subject matter experts from within VA to serve on a Technical Expert Panel that will provide longitudinal oversight of and feedback on our work. The Technical Expert Panel meets every six months and includes approximately 20 national representatives from social work, geriatrics and extended care, primary care, nursing, physical therapy, occupational therapy, pharmacy, health informatics, data analytics, quality improvement and innovation, home- and community-based programs, clinical measurement, and electronic health record standardization and modernization.

#### Implementation team

The implementation team is responsible for implementing the intervention and includes team members from both research and operations. Members of the research team have expertise in geriatrics, program development and implementation, implementation science, partnered research, and project management, while the operational partners have expertise in VA clinical operations and program development and implementation.

#### Evaluation team

The evaluation team is responsible for evaluating the implementation of the intervention. It includes multidisciplinary experts in partnered research, geriatrics, nursing, medical anthropology, implementation science, mixed-methods research, biostatistics, clinical informatics, VA data analysis among older and frail veterans, evaluation of quality initiatives and clinical programs, and age-friendly health systems.

### Description of intervention

The PACT Functional Status Screening Initiative includes four core components, which are described in detail in Table 1. Briefly, these components include: (1) routine, standardized measurement of functional status; (2) annual screening by a licensed vocational nurse (LVN) and follow-up assessment by a primary care provider (PCP); (3) electronic tools and templates to facilitate LVN screening, PCP assessment, and documentation; and (4) reports on functional status that can be tailored to the needs of different sites. The PACT Functional Status Screening Initiative will be implemented among veterans ages 60 and older in primary care settings. We chose this age cut-off because EHR frailty measures identified many younger veterans at risk for functional impairment, and this aligns with geriatrics and primary care priorities to proactively identify veterans at high risk for acute care and skilled nursing facility utilization.

**Table 1:** PACT Functional Status Screening Initiative core components and rationale

1. Routine, standardized functional status measurement
• **Component 1:** Annual measurement with a standardized tool • **Rationale:** Addresses barriers to screening and assessment (competing priorities, lack of standardized processes) and to documenting and using data (lack of standardized data location, poor team coordination)
**2. LVN screening and follow-up PCP assessment**
• **Component 2:** Annual LVN screening during patient triage and follow-up PCP assessment and referral(s), based on score • **Rationale:** Team-based approach clarifies team roles and responsibilities and fosters interprofessional environment
**3. Electronic tools and templates to facilitate LVN screening, PCP assessment, and documentation**
• **Component 3a:** Validated LVN screening tool: (1) brief two-question pre-screener asking about difficulty performing ADLs and IADLs [41], and, among patients who report difficulty on pre-screener, (2) in-depth screener asking about difficulty and needing help with each ADL/IADL [42]. Results used to auto-populate nursing note • **Rationale:** Two-part screener intended to quickly identify patients who would benefit from in-depth screening while “screening out” individuals without impairment, reducing LVN and patient burden. Automation reduces documentation burden
• **Component 3b:** PCP alert and referral menu: If patient screens positive (i.e., reports difficulty/needing help with ≥ 1 ADL/IADL), PCP receives electronic alert linked to referral menu. Alert prompts PCP to review LVN screening results and perform additional assessment as needed. PCP can select up to 5 referrals: physical therapy, occupational therapy, social work, geriatric medicine, prosthetics; referral menus may be adapted at each site to meet local needs and include Community Care referrals • **Rationale:** Addresses clinical partner requests for integration of functional assessment into existing workflows and need for a standardized location to retrieve data on function; alert supports interprofessional approach to measurement; integrated EHR referrals address concerns that data will not be used to inform care and that clinicians lack knowledge of resources, making desired outcome (appropriate referral) more salient for PCPs
**4. Tailored reports**
• **Component 5:** Using Health Factors data, automated reports can be pulled at level of the medical center, clinic, PACT, and/or clinician to report varying statistics (e.g., proportion of veterans needing help with ADLs, specific service referrals, Reach) • **Rationale:** Reports provide access to population-level data to inform strategic planning (e.g., regarding hiring or service capacity needs) and efforts to keep patients functional at home rather than in more costly institutional care

Abbreviations: *ADL* Activities of daily living, *IADL* Instrumental activities of daily living, *LVN* Licensed vocational nurse, *PACT* Patient-Aligned Care Team, *PCP* Primary care provider

## Methods

### Overview of study design

**Fig. 2:**
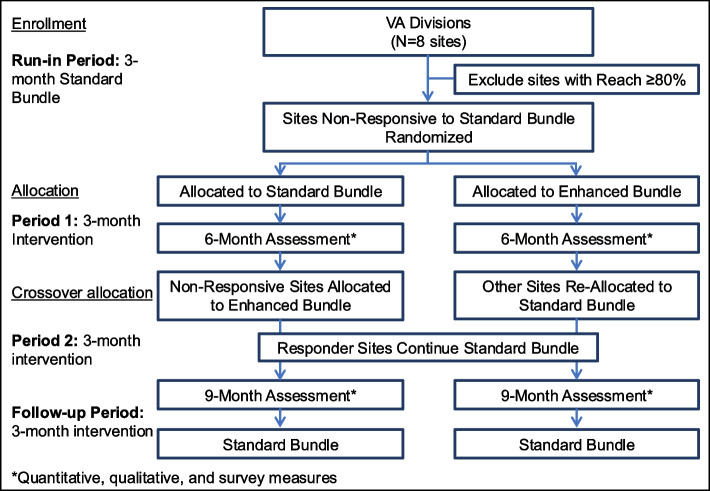
Flow Diagram for Adaptive Cluster-Randomized Controlled Trial. The figure shows the sequence of the four implementation periods, randomization, and cross-over allocation

We will implement and evaluate the PACT Functional Status Screening Initiative using a hybrid type II implementation-effectiveness [43] cluster-randomized adaptive design (Fig. 2) [32–34]. We chose a hybrid type II design because the intervention is supported by promising preliminary data, but the evidence is not yet fully established, and current rates of screening suggest that more intensive implementation strategies may be needed to improve adoption [28]. Because individual implementation sites may have different needs, we will use an adaptive design to determine if more intensive efforts are needed to improve adoption in sites with low uptake. To include an appropriate comparison group, sites with low uptake will be cluster randomized to a standard vs. enhanced bundle of implementation strategies. The design includes a crossover phase so that all of the low uptake sites receive enhanced implementation.

In keeping with our operational partners’ interest in developing proactive approaches to identify high-risk veterans and intervene to prevent or delay functional impairment and associated acute care utilization, we will test the association of clinic-collected functional status measures with automated EHR-based frailty measures. Because frailty typically develops before functional impairment and may be a promising target for intervention [44–47], these findings will clarify how the two approaches might complement and inform one another.

**Fig. 3:**
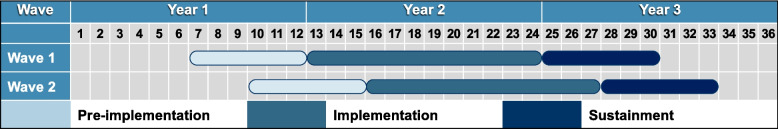
Study Implementation Phases. The figure shows the timeline for the 3 sequential implementation phases and the 2 implementation waves

Based on Primary Care and Office of Nursing Services capacity and input, we will implement the intervention in two waves, with 8 sites total. Sites may range in size depending on organizational capacity (e.g., a full community-based clinic or several primary care teams within a larger site). For both waves, we will cluster at the site level. Each site will move through three phases: Pre-Implementation, Implementation, and Sustainment (Fig. 3) [48].

The PACT Functional Status Screening Initiative was designed for internal VA operational purposes. In 11/2022, all planned procedures were determined by GEC to be operations activities not constituting research and are proceeding without Institutional Review Board review under QUERI authority and oversight according to guidelines set forth in the VHA Program Guide 1200.21 (see Additional file 2).

### Study phases

As shown in Fig. 3 and described below, each site will move through 3 sequential phases: (1) pre-implementation; (2) implementation; and (3) sustainment.

#### Phase 1: Pre-Implementation (6 months)

The Pre-Implementation Phase focuses on: (1) site engagement; (2) identifying potential barriers and proactive adaptations; and (3) developing/finalizing measures and data sources [48].

##### 1. Site engagement

We will meet with operational partners to identify potential sites. We will then meet with sites to present the rationale for the initiative and the core intervention components, including demonstration of electronic tools. We will work with participating sites to identify and prepare clinical champions (a nurse and a PCP). Clinical champions will be added to a Microsoft Teams channel to facilitate ongoing communication and engagement.

##### 2. Identify potential barriers and facilitators to implementation and develop adaptations

At each site, we will conduct one virtual “discussion forum” focus group with 8–10 clinicians per group (60 min). We will use purposeful sampling to include staff roles most involved in practice delivery change (i.e., LVNs, PCPs, social workers) [49]. We will facilitate discussion to understand current processes for measuring function, followed by co-creation of process maps to illustrate the current state [25, 50], and will facilitate brainstorming to elicit barriers at the clinic level. We will also complete individual interviews with two PACT leaders per site (e.g., nurse managers, medical directors; 30 min) to identify implementation barriers and assess degree of leadership support.

We will develop adaptation options based on operational partner priorities and implementation partner feedback [51, 52], such as adaptations related to the frequency of screening or self-administered questionnaires. We will package effective practices in an Implementation Toolkit hosted on the VA Diffusion Marketplace website, including core components, health information technology for the EHR, optional adaptations, and clinical resources.

##### 3. Develop measures and data

Based on our prior work and guided by PRISM, planned implementation outcomes include Reach, Effectiveness, Adoption, Implementation (e.g., fidelity, adaptations), and Maintenance (see also Outcome Measures and Sources by Aim, below). We will refine measures based on insights from operational and implementation partners. We will also use available data (e.g., data generated by the clinical screening tools, data from the Geriatrics and Extended Care Data and Analysis Center) to obtain baseline measures for comparison to the Implementation and Sustainment periods (Tables 2 and 3) [53].

**Table 2:** Implementation outcomes

PRISM/RE-AIM Construct	What we are assessing	Data Sources
**Reach** (primary outcome)	Proportion veterans screened/assessed	CDW
**Adoption** by facility and clinician	Proportion LVNs who complete screening	CDW
	Proportion PCPs who complete assessment	CDW, chart review
	Cognitive participation (knowledge and attitudes related to measuring function)	Survey, qualitative data
**Implementation** • Delivery as intended, fidelity to implementation strategies • Organizational culture • Implementation infrastructure • Recipient perspectives	Organization-level fidelity (≥ 80% checklist items	CDW, qualitative data
	Team dynamics	Survey (PC-TD), qualitative data
	Organizational factors	AES/FEVS
	Staff work experience, engagement	Qualitative data
	Veteran and family caregiver experience	Qualitative data
	Staff & champion experience	Qualitative data
**Maintenance** • Implementation sustained by individuals and facilities over time • Sustainability infrastructure	Reflexive monitoring, cognitive participation, collective action (NPT), team dynamics	Survey (PC-TD), qualitative data
	Sustainment capacity	Survey (CSAT short form)
	Champion attrition (withdrawal, turnover)	Qualitative data
	Completion of and fidelity to Sustainment Plan	Qualitative data
	Leadership support, organizational-level visibility	Qualitative data
**Effectiveness** • Standard vs. enhanced implementation strategy bundle	Proportion of sites with Reach ≥ 80% Recipient perspectives on strategies	CDW, survey, qualitative data

Abbreviations: *AES/FEVS* All Employee Survey/Federal Employee Viewpoint Survey, *CDW* Corporate Data Warehouse, *CSAT* Clinical Sustainability Action Tool, *PC-TD* Primary Care Team Dynamics

**Table 3:** Clinical effectiveness outcomes

**Clinical Effectiveness Outcomes**		Pre-Imp	Implementation				Sustainment	
Measure	Data source		R	P1	P2	P3	3 M	6 M
**Aim 3. Patient-level clinical effectiveness**								
Proportion of Veterans who receive referrals (primary)	CDW	X		X	X	X		X
Facility-free days [54] (secondary)	GECDAC RHF	X		X	X	X		X
Hospitalization, skilled nursing facility utilization, functional status (secondary)	GECDAC Core Files, CDW	X		X	X	X		X
**Aim 4. Effectiveness of EHR-based frailty screening**								
Association of EHR frailty indices with functional status	VA-FI, JEN FI	X		X	X	X		X

Abbreviations: *CDW* Corporate Data Warehouse, *GECDAC* GEC Data & Analysis Center, *RHF* Residential History File, *VA-FI* VA Frailty Index

### Phase 2: Implementation (12 months)

The Implementation periods, shown in Fig. 3, are as follows.

### Run-in period (standard implementation; 3 months)

We will implement the packaged intervention and local adaptations using the standard implementation strategy bundle (coalition building, implementation champions, local technical assistance, system-level audit and feedback). We chose this as the standard approach because its use resulted in 100% Reach in prior work and because use of champions (i.e., individuals who dedicate themselves to supporting and implementing an intervention) are associated with increased uptake of primary care interventions [55–57].

#### Standard implementation strategy bundle

- Build a Coalition: We will cultivate relationships with primary care and nursing leadership at each site, with the goal of increasing leadership awareness and buy-in for implementation efforts. To do so, the Implementation Team will facilitate meetings with site leaders to update them on implementation and elicit their feedback on project challenges to guide implementation efforts.
- Implementation Champions: One nurse and one PCP at each site will serve as Implementation Champions. Champions will help to facilitate problem-solving for the workflow of their site as it relates to implementing the intervention. Champions will receive monthly audit and feedback reports and will be responsible for sharing findings with other clinicians at their site.
- Local Technical Assistance: The Implementation Team will partner with clinicians, champions, and leadership to problem-solve issues related to implementation as they arise. Depending on site needs, additional technical assistance may include one-on-one meetings and weekly office hours to establish feedback channels to report on local progress and enhance buy-in and trust [48].
- System-level Audit and Feedback: Reports on Reach and proportion of patients with functional impairment at each site will be sent monthly by the Implementation Team to Clinical Champions and site leadership (e.g., medical center directors, nurse executives, PACT medical directors, nurse managers).

#### Period 1 (Randomization to Standard or Enhanced Implementation; 3 months)

Our adaptive trial design will deploy resources to sites with the greatest need for enhanced implementation strategies [32–34]. After the Run-In period, data generated by the clinical screening tools (“Health Factors” data) will be used to identify sites with inadequate Reach, defined as < 80% of eligible veterans receiving LVN screening and/or PCP assessment (in keeping with standard benchmarks for adequate adherence to practice guidelines) [58]. We will define PCP assessment as reviewing LVN screening results and either (1) documenting via checkbox that further referral is not needed or (2) placing a referral to address identified impairments. We chose both LVN screening and PCP assessment as the benchmark for implementation response because these combined steps contribute to improved identification (via screening) and management (via assessment/referrals). Sites with ≥ 80% Reach at the end of the Run-In phase will receive the standard implementation bundle for all phases of implementation. For sites with < 80% Reach, we will randomize 1:1 by site to 3 additional months of standard vs. enhanced implementation. Based on current levels of functional status measurement in VA primary care practices (51% measuring function among ≥ 80% of eligible veterans), we anticipate that half of included sites (*n* = 4) will require enhanced implementation. The enhanced implementation strategy bundle will include the components of the standard bundle above (i.e., Coalition Building, Implementation Champions, Technical Assistance) plus clinician-level audit and feedback for participating LVNs and PCPs instead of site-level audit and feedback.

##### Enhanced implementation strategy bundle

- Standard Implementation Strategy Bundle (i.e., Build a Coalition, Implementation Champions, Local Technical Assistance) plus
- Clinician-level Audit and Feedback: Each month, the Implementation Team will e-mail participating LVNs and PCPs a list of their eligible patients who did not receive screening and/or assessment. The report will include a comparison of the percentage of each clinician’s patients who were not screened/assessed relative to other clinicians at that site. Site leadership will continue to receive System-Level Audit and Feedback reports.

#### Period 2 (Crossover Allocation; 3 Months)

At the beginning of Period 2, we will assess Reach and perform crossover allocation. Sites that were initially randomized to standard implementation and are still non-responsive (< 80% Reach) will cross over to enhanced implementation. Sites that were randomized to standard implementation and achieve ≥ 80% Reach will continue to receive standard implementation. Sites randomized to enhanced implementation will receive standard implementation regardless of response.

#### Period 3 (Follow-Up; 3 Months)

All sites will receive standard implementation, and outcomes will be monitored. Calls with champions will focus on developing a Sustainment Plan for each site to hard wire new processes. Champions will consider, “What would be required to make this new process permanent?” Key to supporting Sustainment, during the study, GEC plans to develop an electronic quality measure to report PACT Functional Status Screening Initiative adherence. This will replace the costly and time-intensive External Peer Review Program reporting now in use.

#### Phase 3: Sustainment (6 months)

Implementation support will be withdrawn, and each site will transition to their Sustainment Plan. Implementation and effectiveness outcomes will continue to be evaluated (Tables 2 and 3).

### Outcome measures and sources by aim

#### Aim 1 (Implementation Outcomes)

The primary outcome for Aim 1 is Reach, with secondary outcomes of Adoption, Implementation (fidelity, adaptations), and Maintenance (Table 3). Reach is defined as the proportion of eligible Veterans who receive LVN screening and PCP assessment at each site. We will identify eligible veterans (i.e., ≥ 60 years old, seen in primary care after implementation begins) and use Health Factors to identify completed screening, defined as an LVN completing the electronic tool, and assessment, defined as a PCP reviewing screening results and either (a) documenting via checkbox that further referral is not needed or (b) placing a referral to address impairments.

Adoption is defined as the proportion of LVNs and PCPs at each site who regularly complete screening and assessment. Adequate adoption is defined as ≥ 80% [58]. To identify more nuanced aspects of adoption, we will review a randomly selected subset of charts at each site to examine if clinician notes for the visit when screening was completed have content related to functional status and type of content.

Fidelity to intervention core components will be assessed to determine if core components were implemented as intended, accounting for adaptations and modifications that were consistent vs. inconsistent with intent [48]. Study staff will complete fidelity checklists using electronic health record and qualitative data sources.

Recipient experience will be assessed using periodic reflections for champions, interviews for leadership, veterans, and family caregivers, and surveys for clinicians, and will be used to inform adaptations to intervention components and implementation strategies [59]. To operationalize processes within PRISM’s Adoption, Implementation, and Maintenance domains, we will use Normalization Process Theory (NPT) to guide qualitative data collection [39, 40]. NPT complements PRISM and considers the intervention itself, how staff understand the intervention, attitudes that develop around the intervention, and how it fits into existing roles, systems, and norms. We will use qualitative data to investigate how these processes and constructs shaped implementation and sustainment outcomes.

Maintenance will be measured as continued LVN screening and PCP assessment. We will define adequate maintenance as ≥ 80% at 6 months after beginning Sustainment. To assess clinician capacity for implementation and sustainment, we will use 2 validated survey measures: the short form of the Clinical Sustainability Assessment Tool (CSAT) [60, 61] and Primary Care Team Dynamics (PC-TD) survey [62]. The CSAT short form assesses 7 domains: engaged leadership; engaged clinical partners; organizational readiness; workflow integration; implementation and training; monitoring and evaluation; and outcomes and effectiveness (21 items, Likert scale; higher scores indicate greater capacity for sustainability). The PC-TD subscales measure shared understanding and communication (11 items, Likert scale, higher scores reflect more optimal team dynamics).

To identify potential explanatory factors for outcomes while minimizing burden, we will use existing measures available from administrative data. These include the PACT implementation index score [63], domains of the All Employee Survey (AES; Actions/Behaviors, Climate) [64], and the Federal Employee Viewpoint Survey (FEVS), given to a subset of AES respondents (31 items; domains include employee engagement, burnout) [64]. We will extract pre-post scores for PACT and occupation groups based on available dates most closely aligned with study periods.

#### Aim 2 (Implementation Strategy Effectiveness)

The primary outcome for Aim 2 is Reach, defined as in Aim 1, with ≥ 80% defined as adequate Reach (Table 2). We will assess perspectives on the effectiveness of the standard vs. enhanced implementation strategies using qualitative data.

#### Aim 3 (Clinical Effectiveness)

The primary outcome for Aim 3 is proportion of Veterans with impairments who receive appropriate referrals (Table 3). We will define appropriate referrals as the PCP reviewing the LVN screening results and either (1) documenting via checkbox that further referral is not needed or (2) placing a referral to address identified impairments. We acknowledge that management of functional impairment is complex and there is no “gold standard” for appropriate referrals. For example, a patient may not require a referral because they are already receiving services. Functional status assessment may also inform clinical decision-making (e.g., whether to order cancer screening) but not result in referral. Thus, we will capture clinician judgment of when referrals are appropriate. As noted above, we will use Health Factors data and clinical notes to capture clinician judgement of when referrals are appropriate.

Secondary outcomes include facility-free days, emergency department visits, hospitalization, short- and long-term skilled nursing facility (SNF) use, and functional status. Facility-free days measures the number of days a Veteran is alive and outside a hospital or SNF, calculated from GECDAC residential history files (RHF) [54]. The RHF integrates VA, Medicare (including Medicare Advantage), and Medicaid claims to describe episodes of care for individual Veterans in VA and non-VA settings [65]. We will assess other utilization with GECDAC Core Files [66] and functional status using CDW Health Factors data.

#### Aim 4 (Frailty)

Frailty will be measured using the validated VA Frailty Index (VA-FI), calculated using the cumulative deficit method [67]. The VA-FI includes up to 31 age-related health deficits based on VA EHR diagnostic and procedure codes [46, 68]. Categories include non-frail (0–0.1), pre-frail (0.11–0.2), and frail (> 0.2). Functional status will be measured from Health Factors. In sensitivity analyses, we will examine other EHR frailty indices (e.g., JEN Index [69]).

### Analysis

#### Analysis Plan, Aim 1 (Implementation Outcomes)

We will use descriptive statistics to examine patient- and site-level baseline measures, including outcome measures. For the primary outcome of Reach (0–100%), we will fit three-level mixed effects linear regression models that properly account for the longitudinal design and clustering of patients within sites. Our primary interest is whether baseline characteristics are associated with increased Reach. Inference will be determined using odds ratios and 95% confidence intervals (CIs) and/or *p*-values. A similar strategy will be employed for each secondary outcome.

To analyze Aim 1 qualitative data, we will use rapid analysis methods developed for implementation research that will allow us to use these data in real time to inform implementation strategies [70, 71]. The evaluation team will first develop summary templates with sections corresponding to each PRISM domain and NPT construct. Following each data collection instance, we will summarize key points in the appropriate section of the template; illustrative quotations will be included in summaries. A second team member will review the primary data source and summary for accuracy. We will use matrix analysis to synthesize template data, a method of displaying data to identify relationships between and across units of analysis [70]. Matrices will be organized by site to compare outcomes across sites and type of pre-implementation approach to measurement. Team members will meet regularly to discuss results.

#### Analysis plan, aim 2 (Implementation Strategy Effectiveness)

The primary outcome for Aim 2 is the proportion of sites that achieve ≥ 80% Reach. As in Aim 1, we will first describe site characteristics by implementation strategy bundle (standard vs. enhanced) and baseline proportions of screening and assessment. We will also fit mixed effects logistic regression models where the unit of analysis is the site, accounting for the longitudinal (repeated measures) design and for covariates such as level of champion engagement. Our primary interest is effectiveness of the standard vs. enhanced approach. Inference will be determined using odds ratios and 95% CIs and/or p-values. A similar strategy will be employed for secondary outcomes. We will compare recipient perspectives on the standard vs. enhanced approach using methods as in Aim 1 and triangulate qualitative and quantitative data on effectiveness of strategy bundles [65].

#### Analysis plan, aim 3 (Clinical Effectiveness)

The primary outcome is the proportion of veterans with identified functional impairment(s) who receive appropriate referrals. As in Aim 1, the primary inference for effectiveness (standard vs. enhanced approach) is based on odds ratios and 95% CIs and/or p-values from logistic mixed effects models.

#### Analysis plan, aim 4 (Frailty)

Our primary exposure for Aim 4 is ‘frailty’ operationalized using the VA-FI. We will model the VA-FI as a 3-level categorical measure (i.e., non-frail, pre-frail, frail). Using a similar modeling strategy as for Aims 1–3, we will use an ordinal logistic mixed modeling strategy to estimate the association of clinic-collected functional status measures on frailty (VA-FI; sensitivity analyses with other measures [69]). Inference will be based on estimated odds ratios associated with functional status measures, with associated 95% CIs and/or *p*-values.

#### Missing data and power analysis

For each aim, when missing data exceeds 5% (for outcomes and/or model covariates), in addition to complete case analysis, we will implement and incorporate appropriate missing data methods (e.g., multiple imputation) within each aim’s analytical approach.

Power analysis for the primary clinical effectiveness aim of proportion of veterans receiving appropriate referrals shows that with a moderate effect size of 0.40, α = 0.05, and a design effect of 4.4 to adjust for clustered nature of our data, 80% power can be achieved with a sample of 730 participants. Given the large available sample size (estimated > 5,000 veterans from 8 sites), we have > 80% power to detect a significant difference in our primary clinical effectiveness outcome of proportion of Veterans receiving appropriate referrals.

## Discussion

The overall goal of this project is to determine if implementation of the PACT Functional Status Screening Initiative is associated with increased identification and improved management of functional impairment among older veterans in primary care settings. Our project builds on prior VA efforts to measure functional status using electronic tools administered in primary care. In the current project, we will extend this work by implementing and evaluating an evidence-based, patient-centered, low-burden intervention. Based on prior work, we hypothesize that implementation will reduce burden for primary care teams while leading to increased identification and improved management of functional impairment for older veterans. Furthermore, implementation will provide data to inform VHA strategic planning related to long-term services and supports and implementation of new programs [31]. Specifically, we will generate data to guide VHA’s investment in non-institutional care programs (including home- and community-based services) and allow for targeting and testing of novel programs to address impairments and prevent or delay further functional decline and associated acute care utilization.

The project has notable strengths. These include its focus on functional impairment, an issue that is central to quality of life among older adults and their families and is also costly to the VA and other large health systems [1–8]. The intervention was developed using rigorous methods, including a formative evaluation that identified key determinants of functional status measurement among staff members, including time pressures, cumbersome tools, and the perception that measurement would not be used to inform care [29, 30]. The use of a hybrid cluster-randomized adaptive trial design will allow us to evaluate the impact of the intervention on both implementation and effectiveness measures [32–34]. In addition, the use of the PRISM conceptual framework to guide implementation and evaluation will allow us to systematically consider how the processes of functional status measurement interact with contextual factors in the VA infrastructure and the external environment to influence downstream outcomes. Outcomes will be evaluated using rigorous interdisciplinary mixed methods approaches to provide both quantitative and qualitative measures of implementation and effectiveness. Finally, this project is guided by long-standing collaborative work between our research and operational teams and a shared agenda with operational partners in geriatrics, primary care, and nursing. This partnered approach will maximize the impact of the study’s findings by ensuring that they are incorporated into clinical practice and used to improve care for older veterans.

This project also faces important challenges. One of the most notable is the high level of staff burden and burnout in U.S. primary care settings, related to both time limitations and the growing number of electronic screening tools. Although the current project is intended to reduce burden by streamlining existing screening tools, its focus on screening may still pose a barrier to site recruitment. Ongoing VA efforts to develop electronic screening tools that can be completed by patients or caregivers before an in-person visit may provide a promising alternative platform to disseminate the intervention, which we will explore in our pre-implementation adaptations. Participant burden related to completing evaluation measures (e.g., discussion forums, surveys) may also be an issue. To minimize burden, we have selected brief survey instruments and will use existing data when possible. We will also pilot the survey during Pre-Implementation and reduce its length and/or the frequency of data collection if needed. Based on staff preferences, we may also limit the number of synchronous data collection instances and substitute e-mail or Teams communications. Variability in champion engagement is also possible. We include plans to prepare and engage champions; will measure champion engagement using the fidelity checklist, CSAT, and periodic reflections; and will account for engagement in our analyses. It is also possible that Reach may be lower or higher than expected. If Reach is lower than expected, we will have sufficient capacity to deliver enhanced strategies at all 8 sites. If Reach is higher than expected, this information will help to inform the program design and resources needed for next-stage national implementation of the intervention. Finally, the roll-out of the Electronic Health Record Modernization (EHRM) within VA, which began in 2017, is an important consideration. We will work closely with our operational partners to develop electronic tools for sites that have migrated to the new Federal electronic health record (FEHR), and we will use FEHR data elements to capture outcomes.

### Anticipated contributions to practice

Implementing routine measurement of functional status in primary care has the potential to improve identification and management of functional impairment for older veterans. Improved management includes increasing access to services and supports for veterans and family caregivers, reducing potentially preventable acute care utilization, and enabling veterans to live in the least restrictive setting for as long as possible. Information about the effectiveness of the standard versus enhanced implementation strategies will also inform system-wide implementation efforts, including how to deliver audit and feedback reports at scale (e.g., via dashboards). Implementation will also provide data to inform the delivery of proactive interventions to prevent or delay the development of functional impairment and improve quality of life, health, and independence. In addition, the project will provide key data to inform national VA strategic planning and future system-wide implementation of new programs.

## Supplementary Information

Additional file 1. Standards for Reporting Implementation Studies: the StaRI checklist for completion. File contains completed StaRI checklist for manuscript.

Additional file 2. Determination of Non-Research Activity for “A Partnered Evaluation to Improve Identification and Management of Functional Impairment and Frailty for Older Veterans in Veterans Affairs (VA) Primary Care.” File contains memorandum from GEC confirming that the project meets the criteria for classification of non-research.

Additional file 3. Notification of Funding. File contains formal notice of grant award from QUERI.

## Data Availability

The datasets generated and/or analyzed in the current study are not publicly available due to the identifying nature of data from patients and clinicians who participated in qualitative data collection. Additionally, VA claims data has patient data elements that cannot be shared publicly. However, the methods for data collection and management can be shared publicly, including interview guides and data collection instruments from the corresponding author on reasonable request.

## Abbreviations

ADL: Activities of Daily Living
AES: All Employee Survey
CDW: Corporate Data Warehouse
CI: Confidence Intervals
CSAT: Clinical Sustainability Assessment Tool
EHR: Electronic Health Record
FEVS: Federal Employee Viewpoint Survey
GEC: Geriatrics and Extended Care
GECDAC: Geriatrics and Extended Care Data Analysis Center
IADL: Instrumental Activities of Daily Living
LTSS: Long-Term Services and Supports
LVN: Licensed Vocational Nurse
NPT: Normalization Process Theory
PACT: Patient-Aligned Care Team
PCP: Primary Care Provider
PC-TD: Primary Care Team Dynamics survey
PEI: Partnered Evaluation Initiative
QUERI: Quality Enhancement Research Initiative
RHF: Residential History File
StaRI: Standards for Reporting Implementation Studies
USA: United States of America
VA: Department of Veterans Affairs
VA-FI: Veterans Affairs Frailty Index
VHA: Veterans Health Administration
