# Supplementary material for: Study protocol: type II hybrid effectiveness-implementation study of routine functional status screening in VA primary care
Source: Implement Sci Commun. 2025 Jan 31;6:15. doi: 10.1186/s43058-025-00698-w (PMC11786338; doi:10.1186/s43058-025-00698-w)
Supplement: Supplementary file 2 — Additional file 2. Determination of Non-Research Activity for “A Partnered Evaluation to Improve Identification and Management of Functional Impairment and Frailty for Older Veterans in Veterans Affairs (VA) Primary Care.” File contains memorandum from GEC confirming that the project meets the criteria for classification of non-research. [file 43058_2025_698_MOESM2_ESM.pdf]

Department of Veterans Affairs

Memorandum

Date: November 30, 2022

From: Office of Geriatrics and Extended Care (12GEC)

Subj: Determination of Non-Research Activity for "A Partnered Evaluation to Improve Identification and Management of Functional Impairment and Frailty for Older Veterans in Veterans Affairs (VA) Primary Care"

To: Rebecca Brown and Francesca Nicosia, Principal Investigators

1. The purpose of this letter is to confirm that the project *"A Partnered Evaluation to Improve Identification and Management of Functional Impairment and Frailty for Older Veterans in VA Primary Care"* meets the criteria for classification as non-research. This project is being conducted at the Corporal Michael J. Crescenz VA Medical Center, San Francisco VA Healthcare System, Canadaigua VA Medical Center, VA Boston Health Care System, and VA Indiana Healthcare System, and is led by Drs. Rebecca Brown and Francesca Nicosia. The implementation/evaluation is designed to inform quality improvement efforts as part of the agreed-upon protocol established by the VA Office of Research and Development Program Guide 1200.21.

2. The purpose of this project is to support internal efforts to 1) implement routine functional status measurement among older Veterans in primary care; and 2) evaluate the implementation of this intervention. The goal of this work is to 1) increase identification of functional impairment among older Veterans; 2) improve management of functional impairment among older Veterans; 3) determine the most effective way to implement measurement; and 4) determine if frailty measures can identify Veterans at risk for developing functional impairment. The project will involve 1) use of secondary and primary VA data collected using documentation that is part of routine care and/or clinical management along with Program Office data when applicable; 2) quantitative data collection using surveys administered to VA staff; and 3) qualitative data collection among Veterans and VA staff. This project will use information that is designed for quality improvement initiatives, as described in Program Guide 1200.21, for the purposes of program implementation/evaluation.

3. These activities are designed and implemented for internal VA purposes and findings are intended to be used to better inform care in VA. This project is not designed to inform activities beyond VA, produce information that expands the knowledge base of a scientific discipline or other scholarly field, and does not involve collecting additional data or performing analyses that are not needed for the purposes of this internal implementation.

Subj: Determination of Non-Research Activity for “A Partnered Evaluation to Improve Identification and Management of Functional Impairment and Frailty for Older Veterans in VA Primary Care”

4. We will disseminate findings from this project to advance GEC’s mission to improve the health and well-being of older Veterans.

CHERYL SCHMITZ

Digitally signed by CHERYL SCHMITZ
Date: 2022.11.30 14:09:28 -05'00'

Cheryl Schmitz, M.S., RN, CNS-BC, NE-BC
Deputy Executive Director, Office of Geriatrics and Extended Care
