# Supplementary material for: Study protocol: type II hybrid effectiveness-implementation study of routine functional status screening in VA primary care
Source: Implement Sci Commun. 2025 Jan 31;6:15. doi: 10.1186/s43058-025-00698-w (PMC11786338; doi:10.1186/s43058-025-00698-w)
Supplement: Supplementary file 3 — Additional file 3. Notification of Funding. File contains formal notice of grant award from QUERI. [file 43058_2025_698_MOESM3_ESM.pdf]

DEPARTMENT OF VETERANS AFFAIRS
Health Services Research
811 Vermont Ave, NW
Washington, DC 20420

October 1, 2023

Philadelphia VA Medical Center
3900 Woodland Ave
Philadelphia, PA 19104

SUBJECT: Funding Notification
Quality Enhancement Research Initiative (QUERI)
Project #: PEC 23-072
Title: A Partnered Evaluation to Improve Identification and Management of Functional Impairment and Frailty for Older Veterans in VA Primary Care
Principal Investigators: Rebecca Brown, MD; Francesca Nicosia, PhD

1. **Funding Decision**. I am pleased to notify you that the Quality Enhancement Research Initiative (QUERI) has approved funding for the project described above. Acceptance of this funding acknowledges agreement to comply with VA policies regarding intellectual property disclosure obligations and ownership rights resulting from this work. Funding will begin on **October 1, 2023**.

2. **Budget Information**. We have asked the Office of the Chief Financial Officer, Allocation Control Service, to transfer Program 870 funds to your facility for **FY24** with plans for funding through **September 30, 2026** as detailed in the enclosed budget documents (**Enclosure 1**).

3. **Reporting Requirements**. HSR/QUERI requires copies of all publications based on the QUERI-funded work. Approval of future HSR/QUERI funding is contingent on the investigator's adherence to these critical requirements. For additional information and details regarding investigator reporting requirements, please consult your local R&D office.

   a. Publication Transmittal. Investigators are required to promptly notify HSR/QUERI of all publications resulting from QUERI-funded work. Submit your notification as soon as it is accepted for publication, by following the steps below:
      - Go to the PubTracker Website by copying and pasting the following URL into your browser: http://vaww.pubtracker.research.va.gov/PubTracker/default.cfm (Access restricted to VA Intranet using Internet Explorer)
      - Select the appropriate submission type from the "New Pre-pub Notification" and fill in the form (Be sure to upload a copy of the complete accepted article or presentation abstract).

4. **VA Acknowledgment**. Each publication, press release or other document that cites results from VA-supported research must include an acknowledgment of VA support using the eRA application number or project ID number, such as "The project described was supported by (type of award, e.g., Merit Review, Career Development Award, Pilot Project) Award Number ___________ from the United States (U.S.) Department of Veterans Affairs Health Services Research & Development Service of the VA Office of Research and Development." When the work was solely funded by VA, authors must list their VA affiliation first. When the author also holds a faculty appointment, the academic title and school also may be acknowledged. All publications should include a disclaimer similar to this statement: "The views expressed in this article are those of the author(s) and do not necessarily represent the views of the Department of Veterans Affairs."

5. **Communications**. You may direct questions regarding this project to vacoqueri@va.gov. If, while conducting this project, you encounter any research/quality improvement barriers that HSR/QUERI should be notified about and/or may assist in resolving, they should be forwarded to HSR by the COIN AO or Research AO via the following link: http://vaww.hsrd.research.va.gov/research-barriers/. Please include the project number (above) in any communication concerning this project. Please be reminded that all communication regarding this project should go through the local research office. The principal investigator is responsible for relating all communications from VA Central Office to any co-investigators, as necessary.

Melissa Braganza, MPH
Acting Director, QUERI
VHA HSR (14RDH)

Enclosure: Budget Sheet
